# Advanced Parkinson's disease effect on goal-directed and habitual processes involved in visuomotor associative learning

**DOI:** 10.3389/fnhum.2012.00351

**Published:** 2013-01-16

**Authors:** Fadila Hadj-Bouziane, Isabelle Benatru, Andrea Brovelli, Hélène Klinger, Stéphane Thobois, Emmanuel Broussolle, Driss Boussaoud, Martine Meunier

**Affiliations:** ^1^INSERM U1028, Lyon Neuroscience Research Center, IMPACT TeamLyon, France; ^2^CNRS UMR5292, Lyon Neuroscience Research Center, IMPACT TeamLyon, France; ^3^University Lyon 1Lyon, France; ^4^Service de Neurologie, CHU PoitiersPoitiers, France; ^5^Institut de Neurosciences de la Timone (INT), UMR 7289 CNRS and Aix-Marseille Université, Campus Santé TimoneMarseille, France; ^6^Hospices Civils de Lyon, Hôpital Neurologique Pierre Wertheimer, Service de Neurologie CLyon, France; ^7^Centre de Neurosciences Cognitives, CNRS UMR 5229Bron, France; ^8^Institut de Neuroscience des Systémes, UMR 1106, INSERM, Aix-Marseille UniversitéMarseille, France

**Keywords:** feedback-based learning, striatum, habits, goal-directed actions, Parkinson's disease

## Abstract

The present behavioral study re-addresses the question of habit learning in Parkinson's disease (PD). Patients were early onset, non-demented, dopa-responsive, candidates for surgical treatment, similar to those we found earlier as suffering greater dopamine depletion in the putamen than in the caudate nucleus. The task was the same conditional associative learning task as that used previously in monkeys and healthy humans to unveil the striatum involvement in habit learning. Sixteen patients and 20 age- and education-matched healthy control subjects learned sets of 3 visuo-motor associations between complex patterns and joystick displacements during two testing sessions separated by a few hours. We distinguished errors preceding vs. following the first correct response to compare patients' performance during the earliest phase of learning dominated by goal-directed actions with that observed later on, when responses start to become habitual. The disease significantly retarded both learning phases, especially in patients under 60 years of age. However, only the late phase deficit was disease severity-dependent and persisted on the second testing session. These findings provide the first corroboration in Parkinson patients of two ideas well-established in the animal literature. The first is the idea that associating visual stimuli to motor acts is a form of habit learning that engages the striatum. It is confirmed here by the global impairment in visuo-motor learning induced by PD. The second idea is that goal-directed behaviors are predominantly caudate-dependent whereas habitual responses are primarily putamen-dependent. At the advanced PD stages tested here, dopamine depletion is greater in the putamen than in the caudate nucleus. Accordingly, the late phase of learning corresponding to the emergence of habitual responses was more vulnerable to the disease than the early phase dominated by goal-directed actions.

## Introduction

The idea that habits depend on the striatum has been around for a long time. It emerged in the 1960s when it became obvious that some forms of learning were spared by the dense amnesia induced by medial temporal removal in patient H.M. (Seger and Spiering, 2011). Mishkin and colleagues were the first to formalize it (Mishkin and Petri, 1984; Mishkin et al., 1984). They defined habits as stimulus–response connections formed gradually through trial and error on the basis of reinforcement. They hypothesized that forming new habits requires the striatum as memorizing facts and events requires the medial temporal lobe (Turchi et al., 2010). This implied that habits should eventually be wiped out by Parkinson's disease (PD) as radically as facts and events are lost in amnesia. It took 12 years and a very difficult probabilistic stimulus-response task to start getting evidence supporting this claim (Knowlton et al., 1996; Frank et al., 2004; Shohamy et al., 2004a). In addition, with mild to moderate PD, patients were impaired but managed to improve over days (Frank et al., 2004; Shohamy et al., 2004b).

Then, the learning theory literature proposed to distinguish two separate habit processes: the outcome-driven goal-directed actions mediating acquisition of novel stimulus–response associations and the stimulus-driven habitual responses mediating the subsequent stabilization of newly formed bonds (Dickinson, 1985; Rescorla, 1991; Dickinson and Balleine, 1993, 1994; Dickinson, 1994; Staddon and Cerutti, 2003; Balleine and O'Doherty, 2010; Daw et al., 2011; Doll et al., 2012). These two processes are viewed as anatomically segregated within the rat striatum. Goal directed actions would depend preferentially on the rat's posterior dorsomedial striatum and habitual responses on the dorsolateral striatum (Reading et al., 1991; Packard and McGaugh, 1996; Ragozzino et al., 2002; Yin et al., 2005a,b, 2006; Stalnaker et al., 2010; Thorn et al., 2010; but see Stalnaker et al., 2010). A similar segregation may hold true for analogous regions in the primate brain, the caudate nucleus/rostral putamen, and caudal putamen regions, respectively (Miyachi et al., 1997, 2002; O'Doherty et al., 2004; Tricomi et al., 2009; Wunderlich et al., 2012a). In PD, degeneration of the dopaminergic pathways is thought to affect caudal putamen earlier and more severely than the rostral putamen/caudate complex (Redgrave et al., 2010). The new model on the striatum role in habits therefore predicts a disproportional effect of PD on habitual responses. History repeats itself, however, evidence supporting this new claim is not coming easily. Using a conflict task based on the new model, De Wit et al. (2011) tested patients with mild PD and unexpectedly found only a disease severity-dependent deficit in goal-directed actions.

Here we built on our extensive experience of conditional visuo-motor learning to readdress the question of the habit deficit associated with PD. Conditional visuo-motor learning consists in mapping, through trial-and-error, a set of stimuli onto a set of actions according to purely arbitrary rules (Petrides, 1985a,b). It has extensively been used to demonstrate the role of the dorsal striatum in habit learning in both humans and animals using different techniques (electrophysiology in rats: Carelli and Deadwyler, 1997; Jog et al., 1999; Barnes et al., 2005; Tang et al., 2007; Kim et al., 2009; Kimchi et al., 2009; electrophysiology in monkeys: Tremblay et al., 1998; Hadj-Bouziane and Boussaoud, 2003; Brasted and Wise, 2004; Pasupathy and Miller, 2005; Buch et al., 2006; Williams and Eskandar, 2006; Histed et al., 2009; neuropsychology in humans and monkeys: Petrides, 1985b, 1997; Wise et al., 1996; Passingham et al., 2000; Bussey et al., 2001; Nixon et al., 2004; neuroimaging in humans: Deiber et al., 1997; Toni and Passingham, 1999; Toni et al., 2001a,b; Simon et al., 2002; Haruno et al., 2004; O'Doherty et al., 2004; Tanaka et al., 2004; Tricomi et al., 2004; Boettiger and D'Esposito, 2005; Delgado et al., 2005; Law et al., 2005; Grol et al., 2006; Haruno and Kawato, 2006; Pessiglione et al., 2006; Brovelli et al., 2008; Tricomi et al., 2009).

Using single-cell recordings, we identified two distinct neuronal populations within the monkey striatum. One presents a transient increase in firing rate very early on during learning of novel associations, presumably reflecting the substrate for goal-directed actions. The other presents, on the opposite, a protracted increase in firing rate paralleling performance improvement and stabilizing late in learning, presumably reflecting the substrate for habitual responses (Hadj-Bouziane and Boussaoud, 2003; Hadj-Bouziane et al., 2006). Later, the first type of neurons was found to predominate in the monkey caudate nucleus, whereas the second type was more frequent in the putamen (Williams and Eskandar, 2006). In addition, we recently scanned healthy humans with fMRI on the same conditional visuo-motor task and found positive evidence of a differential involvement of the caudate nucleus and putamen in, respectively, the early vs. late learning of conditional visuo-motor associations (Brovelli et al., 2011; Amiez et al., 2012).

The present behavioral study in PD patients complements our previous approaches in monkeys and healthy humans. Its aim was to distinguish for the first time the effects of PD on performance during the early vs. late learning phases of visuo-motor associations. Conditional learning has been tested in PD patients previously but yielded inconsistent results. Learning of visuo-visual or visuo-verbal associations was often found disrupted, but not always (see respectively, Gotham et al., 1988; Taylor et al., 1990; Vriezen and Moscovitch, 1990; Sprengelmeyer et al., 1995; Pillon et al., 1998; and Marie et al., 1999 vs. Canavan et al., 1989; Postle et al., 1997; Bedard and Sanes, 2009). Learning of visuo-motor associations was studied only once, and no deficit could be detected in the sample of patients tested (Canavan et al., 1989). All these previous studies used a correction procedure that made it impossible to study early vs. late learning separately. As in Petrides (1985a,b), an incorrect response was followed by re-presentation of the same stimulus as many times as it took to get a correct answer. Here, we applied to Parkinson's patients the protocol devoid of correction procedure that we successfully used earlier in monkeys and healthy humans to reveal the striatum involvement in habit learning (monkeys: Hadj-Bouziane and Boussaoud, 2003; Hadj-Bouziane et al., 2003, 2006; humans: Simon et al., 2002; Brovelli et al., 2008, 2011; Amiez et al., 2012).

Sixteen patients and 20 age-matched controls had to learn, by trial and error, the associations between 3 visual stimuli and 3 joystick movements. Learning was pursued until a criterion of three consecutive correct responses was achieved for each association. Performance was then analyzed on a trial-by-trial basis for each association separately. The number of errors committed prior to the first correct response was used to measure the early learning phase dominated by goal-directed actions, and the number of errors committed after the first correct response up to the achievement of the learning criterion was used to measure late learning and the emergence of habitual responses. In healthy subjects, the dorsal striatum is activated, together with the dorsal fronto-parietal network and the ventrolateral prefrontal cortex, both on the incorrect and first correct trials and may reflect the processing of relevant visuomotor mappings during the early phases of learning (Brovelli et al., 2008) whereas subsequent repetitions of the correct response activate the putamen (Amiez et al., 2012).

First, given the large body of evidence that conditional visuo-motor learning engages the striatum in healthy animals and humans, we expected the learning performance of the patients to be markedly retarded. Second, based on the assumptions that goal-directed behaviors are predominantly caudate-dependent whereas habitual responses are primarily putamen-dependent, we predicted that habitual responses would be particularly impaired in our population of PD patients, for which we found earlier greater dopamine depletion in the putamen than in the caudate nucleus (Broussolle et al., 1999).

## Methods

### Participants

The study was approved by the local Human Research Ethics Committee and all subjects gave informed consent. Sixteen non-demented and non-depressed patients with advanced PD and 20 control subjects, without neurological or psychiatric history and matching the patients for age and level of education, participated to the study (Tables 1 and 2). All PD patients were under consideration for surgical treatment in the Department of Neurology C of the Pierre Wertheimer Neurological Hospital, Lyon, France. They all fulfilled the UK Parkinson's Disease Society, Brain Bank (UKPDSBB) diagnostic criteria (Gibb and Lees, 1988). They presented an akinetorigid syndrome, resting tremor, or both, as well as a good sensitivity to levodopa treatment, marked dyskinesias and on–off fluctuations. The Beck Depression Inventory and Mattis dementia scale were administered (on medication) to 13/16 patients (Table 2) and a full neuropsychological evaluation including the Starkstein apathy scale, the Grober and Buschke memory test and the Modified Wisconsin Card Sorting Test was available for 10/16 patients.

**Table 1:** **Demographic details of the control subjects matching patients for age and education level (means ± SD)**.

**Case**	**Sex**	**Age (years)**	**Education (years)**
1	F	57	12
2	M	58	14
3	F	51	9
4	M	52	9
5	F	55	12
6	F	56	14
7	M	48	12
8	F	66	9
9	F	72	14
10	M	71	12
11	M	63	14
12	F	65	9
13	F	54	9
14	M	60	9
15	M	47	12
16	F	46	9
17	M	46	12
18	M	65	19
19	F	60	14
20	M	44	19
Mean	10M/	56.8	12.2
SD	10F	8.4	3.1

Cases *1–11* were tested only once, whereas cases *12–20* were, like patients, tested twice.

**Table 2:** **Demographic and clinical details of the patients (means ± SD)**.

**Case**	**Sex**	**Age (years)**	**Education (years)**	**Disease duration (years)**	**Hoehn-Yahr on(/5)**	**Hoehn-Yshr off(/5)**	**Dose equ.Ldopa (mg/j)**	**Beck Depression Invent.(/63)**	**Mattis Dementia scale (/144)**
1	M	60	9	10	2	3	1200	33	128
2	F	43	9	12	2.5	3	500	21	141
3	F	50	12	13	2	3	1125	10	138
4	M	52	9	13	2	4	1775	10	132
5	M	62	9	7	1.5	3	1290	6	124
6	M	49	12	9	1.5	3	1260	21	142
7	M	53	9	11	2	2.5	1175	11	134
8	M	67	10	10	2	3	1150	10	138
9	M	53	10	11	2	4	1500	14	133
10	M	54	19	10	1	3	1500	9	141
11	M	51	14	15	2	4	900	5	134
12	F	67	9	13	2	3	1175	15	130
13	F	61	9	12	3	5	1400	–	–
14	M	61	9	12	2	3	1200	2	135
15	F	40	14	10	2	3	600	–	–
16	M	61	19	9	1.5	2.5	1000	–	–
Mean	11M/	55.2	11.3	11.1	1.9	3.25	1172	13	135
SD	5F	7.9	3.5	2	0.4	0.6	320	8	5

All patients underwent two testing sessions, one with and one without medication. Patients *1–8* were tested off medication first, patients *9–16* were tested on medication first. Daily doses of dopaminergic treatment were expressed as levodopa equivalent dose in mg/24 h using a common formula: 100 mg levodopa = 10 mg bromocriptine = 6 mg ropinirole = 1 mg pergolide = 1 mg lisuride = 1.2 mg pramipexole = 50 mg piribedil. Patients UPDRS III scores averaged 11/108 on medication (SD 4) and 40/108 off medication (SD 10).

Patients underwent two testing sessions (hereafter referred to as test and retest), lasting about 1 h each, and carried out 3–5 h apart on the same day. After an overnight withdrawal of medication performed for clinical purposes, patients were either tested off medication first, and then on medication (PD-1 subgroup, cases 1–8) or the reverse (PD-2 subgroup cases 9–16). The on medication state was defined as 1 h after an acute challenge of levodopa corresponding to 1.5 × the patient's current dose. The off and on medication state were evaluated based on both clinical examination and UPDRS and Hoehn & Yahr scales. The control group initially comprised 11 subjects submitted to a single session (cases 1–11). As results revealed a marked test-retest effect in patients, 9 more controls (cases 12–20) were added who, like patients, underwent two testing sessions separated by 3–5 h.

### Procedure

**Figure 1:**
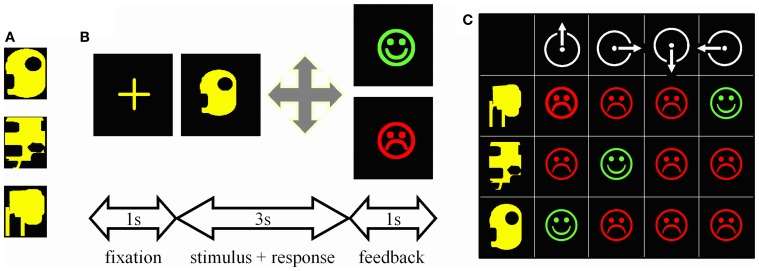
**Experimental task. (A)** One of the trios of complex geometric patterns used as stimuli. **(B)** Trial time-course. First, a central cross appeared at the center of the screen for 1 s. Then, one stimulus was presented for 3 s during which the subject had to guess which among the four possible joystick displacements was associated with this stimulus. A correct response was followed by a green happy face, an incorrect one by a red sad face. Both feedbacks were presented for 1 s. **(C)** Matrix summarizing the 12 possible associations to be tested when mapping 3 stimuli (rows) onto 4 joystick displacements (columns). Subjects can try associations randomly and memorize each stimulus—response—outcome link thus explored; such an “unorganized” way to solve the task was not infrequent in controls. Alternatively, given that each stimulus appeared once per block of three trials, one can select a movement, repeat it for every stimulus, and then reiterate the procedure for the next movement on the next block of 3 trials. This way the three correct associations are systematically found within the 12 trials represented in the matrix; PD patients particularly relied on this optimal learning strategy.

Stimuli were computer-generated colored geometric patterns presented at the center of a computer screen (Figure 1A). There were four possible motor responses: the right, left, forward, or backward displacement of a joystick. During each trial (Figure 1B), a central cross was presented for 1 s, the stimulus for 3 s, and the feedback for 1 s; a green happy face signaled a correct response, a red sad face an incorrect one. Subjects had to make a response during the 3s-stimulus presentation otherwise the trial was aborted and a large red cross presented for 1 s.

Subjects mapped 3 visual stimuli onto 3 joystick movements. Stimuli varied in shape, but were of the same overall size and color (Figure 1A). All 3 stimuli were presented once before they were presented again in a new random sequence. In other words, on the first trial, one of the 3 stimuli was randomly selected and presented; on the second trial, one of the remaining two stimuli was randomly selected and presented, regardless of the outcome of the previous trial, and then the remaining one of the 3 stimuli was presented. Testing continued like this until the subject reached the criterion of three consecutive correct responses for each stimulus. Even if two stimuli were learned, presentation of all three stimuli was pursued until the last stimulus was learned too. If patients failed to learn, however, testing was interrupted after 50 trials to minimize their discomfort. Six different trios of stimuli were learned in this way, 3 during the test, and 3 during the retest. The order of presentation of the six trios during test and retest was fixed and identical across subjects.

As each stimulus appeared once per block of three trials, the optimal way to search for the correct association was to select one movement and repeat it until it proves either correct or not associated with any stimulus (Figure 1C). Subjects were never informed about this optimal movement-oriented strategy, but each of them was asked at the end of the experiment to describe the way he/she used to solve the task. Of the 36 subjects tested, only one, a patient, reported having tried each movement in turn in a clockwise manner.

### Data analysis

We calculated five variables: (1) global scores, i.e., the total number of trials and errors required to reach, or fail to reach criterion for each trio of associations, (2) response times, i.e., the time elapsed between stimulus onset and the subject's response, (3) search errors, i.e., errors made before the first correct response, (4) repetition errors, i.e., errors made after the first correct response and before the learning criterion or the limit of 50 trials, and (5) strategy scores i.e., number of repetition of the same movement after an incorrect response, expressed as a percentage of the total number of errors to criterion. Strategy scores under 30 reflects rare use of the optimal strategy consisting in repeating the same movement until it proves correct, scores above 60 to a quasi-systematic use.

We used 2 × 2 ANOVAs with group and session, Student's two-sample *t*-tests for separate variance and paired *t*-tests to assess between- and within-groups differences. The *p*-value for significance of these *post-hoc t*-tests was set at a Bonferroni-adjusted *p* < 0.025. Comparisons involving retest involve only the 9 controls that were tested twice like patients, whereas comparisons performed on test involved all 20 controls. In controls, Pearson's correlation analyses were used to determine whether age and strategy use predicted learning scores on test (these analyses were not performed on retest due to the small number of subjects involved). In patients, Pearson's correlation analyses were used to determine the influence of age, strategy use, disease stage, and neuropsychological variables on learning scores, and these analyses were performed on both test and retest.

## Results

The 20 controls matched the 16 PD patients for age (*t*_34_ = 0.6, *p* = 0.58), and education level (*t*_34_ = 0.7, *p* = 0.48). Patients' response times were expectedly longer than controls reaching 1519 ± 344 ms on test and 1507 ± 108 ms on retest compared to 1286 ± 233 ms and 1118 ± 60 ms, respectively, in controls (group effect: *F*_(1, 23)_ = 8.4, *p* = 0.008; session effect and interaction, *F*s ≤ 0.4, *ps* > 0.51). They remained, however, well under 3 s, indicating that the learning deficit described below was not due do a motor inability to respond within the imparted time.

More surprising was the lack of effect of medication. As summarized in Table 3, in both the PD-1 (test off–retest on) and PD-2 (test on–retest off) subgroups, most conditional learning measures improved slightly on levodopa relative to off levodopa. Yet, none of these on/off differences reached significance whether the PD-1 and PD-2 subgroups were considered separately or together. The results below therefore focus on the session effect (test vs. retest), which, unlike medication, markedly affected patients' performance.

**Table 3:** **Age, education level, disease stage, and conditional learning scores (mean ± sem) of patients according to medication status**.

	**PD-1 (*n* = 8)**		**PD-2 (*n* = 8)**	
	**Mean**	**SEM**	**Mean**	**SEM**
Age (years)	54.5	2.8	56	3
Education (years)	9.9	0.5	12.9	1.5
Hoehn & Yahr off (/5)	3.1	0.1	3.4	0.3
**TEST**	**OFF**		**ON**	
Reaction times (ms)	1601	136	1438	107
Trials per set (max: 50)	39.9	2.9	33.2	4.2
Search errors	13.2	2	6.7	1.2
Retention errors	10.5	2.2	9.2	3.3
Strategy score	14.8	4.7	24.6	5.8
**RETEST**	**ON**		**OFF**	
Reaction times (ms)	1482	170	1532	143
Trials per set (max: 50)	28	3.9	30	3.7
Search errors	8.1	2.5	6.6	1.4
Retention errors	5.8	2.2	6.5	2.7
Strategy score	25.3	6.2	34.7	7.3

The counterbalanced within-subject design revealed no reliable effect of the acute challenge of levodopa.

### Global conditional learning performance

The results are detailed for trials to criterion; unless otherwise indicated, errors to criterion yielded the same conclusions.

#### Global impairment and disease stage

**Figure 2:**
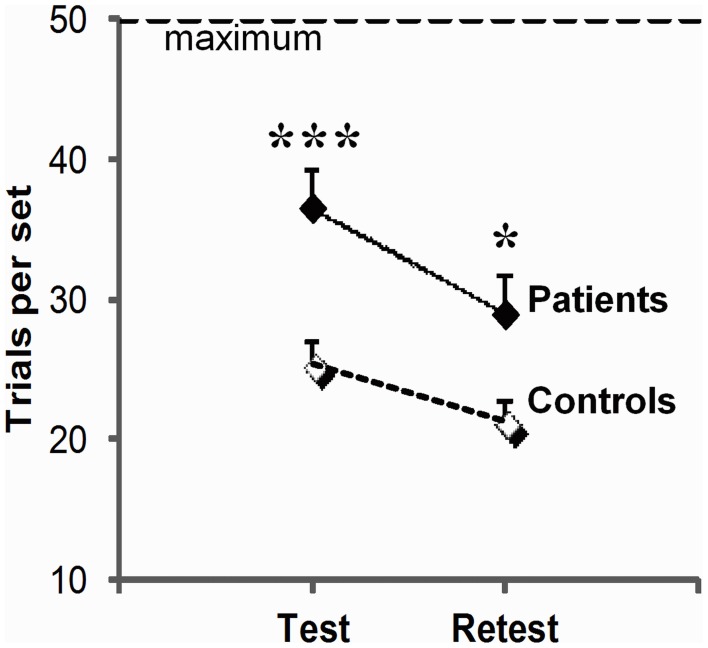
**Global learning impairment in PD patients relative to controls.** Number of trials (trials per set, mean, and SEM) to reach or fail to reach criterion of three consecutive correct responses for all 3 associations within the imparted 50 trials (maximum) on the test and retest session; the higher the scores, the worse the performance. For each session, scores were averaged over three trios of visuo-motor associations (^***^p = 0.001; ^*^p = 0.05: between group differences as revealed by two-sample *t*-tests).

As illustrated in Figure 2, patients systematically performed more poorly than controls (group effect, *F*_(1, 23)_ = 5.8, *p* = 0.02) although, like controls, they improved across sessions (session effect *F*_(1, 23)_ = 13.5, *p* = 0.001; group × session interaction, *F*_(1, 23)_ = 0.5, *p* = 0.83 ns). They required 44% more trials per set on test (*t*_34_ = 3.8, *p* = 0.001), and 36% more trials per set on retest (*t*_23_ = 2.1, *p* = 0.02). More advanced disease stage as evaluated by Hoehn & Yahr on medication scores was correlated with more trials per set on test (test: *r* = 0.50, *p* = 0.05), and a combination of more trials per set (*r* = 0.65, *p* = 0.006), more errors per set (*r* = 0.67, *p* = 0.005) and poorer strategy scores on retest (*r* = −0.53, *p* = 0.03). We did not find reliable correlation between patients' impairment on conditional learning and their neuropsychological scores, but this negative result should be taken with caution as a complete neuropsychological evaluation could only be obtained from only 10 of our 16 patients.

#### Potential influence of age and strategy

**Figure 3:**
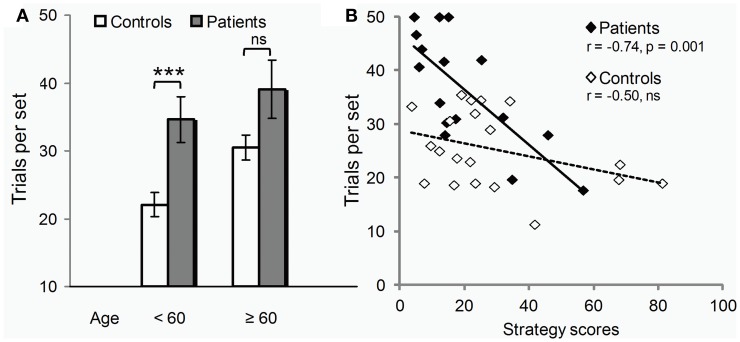
**Age class and strategy use influence on test. (A)** Under 60 years of age (12/20 controls, 9/16 patients), PD deleterious effect on learning (trials per set, i.e., trials required to learn all 3 associations to criterion, mean ± SEM) was clear-cut (two-sample *t*-tests: ^***^p = 0.006). Beyond 60 years of age, the disease effect was only marginally worse than that of normal aging (two-sample *t*-tests: n.s., non significant *p* = 0.10). **(B)** Irrespective of age, PD effect on learning (y axis, trials per set) varied across individuals depending on their ability to use the optimal learning strategy (strategy scores, x-axis) as revealed by Pearson correlation analyses (*r*'s and *p*'s).

Controls seemed more vulnerable to aging than patients (Figure 3A). Only in controls, were scores positively correlated with age (errors: *r* = 0.46, *p* = 0.04 compared to *r* = 0.13, *p* = 0.63 ns in patients). Also, only in controls, did performance deteriorate after 60 years of age (*t*_18_ = 3.2, *p* = 0.005; patients: *t*_14_ = 0.8, *p* = 0.43 ns). The global impairment reported above was thus due largely to the younger subjects of the two groups (*t*_19_ = 3.5, *p* = 0.006), the difference between older subjects being marginal (*t*_13_ = 1.9, *p* = 0.10).

Conversely, performance was significantly correlated with strategy scores only in patients (errors: *r* = −0.71, *p* = 0.002; compared to: *r* = −0.31, *p* = 0.18 ns in controls). As illustrated in Figure 3B, patients with high strategy scores performed within controls' range. Most used the strategy without being aware of it. The one patient who used it deliberately, trying each movement in turn in a clockwise order, outperformed all other patients, and all controls but the youngest one.

#### Summary

As a group, the 16 early onset PD patients tested at advanced stages of the disease in the present study were unequivocally impaired in conditional visuo-motor learning. At individual level, however, the magnitude of this impairment varied with age and the ability to use the optimal learning strategy. Clear-cut in younger patients, the deficit became indistinguishable from the decline produced by normal aging in older patients, and irrespective of age, could be totally overcome by strategy use.

### Early vs. late learning

#### Search vs. repetition errors and disease stage

**Figure 4:**
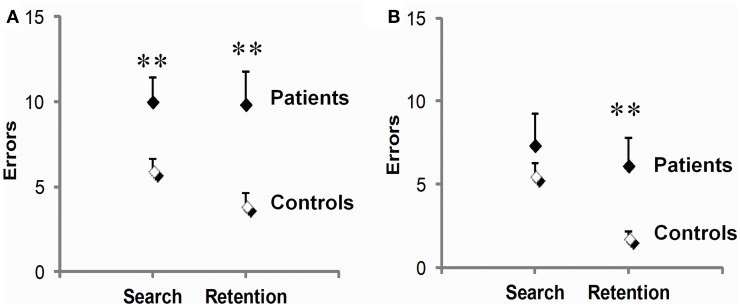
**Early (search errors) and late (repetition errors) learning deficits in PD patients relative to controls.** Patients were impaired on both measures on test **(A)**, but not on retest **(B)**, where only their repetition errors were significantly increased. Scores are means and SEM per set of three visuo-motor associations averaged over three sets. ^**^p ≤ 0.02, two-sample *t*-tests.

On test (Figure 4A), PD patients were significantly impaired during both the early phase preceding the first correct response and the late phase following it. They made 66% more search errors (*t*_34_ = 2.6, *p* = 0.02), and 152% more repetition errors than controls (9.9 ± 1.9 vs. 3.9 ± 0.8; *t*_34_ = 2.9, *p* = 0.01). On retest (Figure 4B), a dissociation between these two deficits emerged. There, patients' early phase deficit was no longer significant (*t*_23_ = 1.2, *p* = 0.25), whereas their late phase deficit persisted, amounting to a marked 237% increase in repetition errors relative to controls (*t*_23_ = 2.5, *p* = 0.02). Another difference between the two deficits concerned their link with disease stage as evaluated by Hoehn & Yahr on medication scores. Disease stage reliably predicted repetition errors (*r* = 0.64, *p* = 0.007, on test and *r* = 0.70, *p* = 0.003, for retest), but not search errors (*r* = 0.02 on test and *r* = 0.39 for retest, *p*'s ns).

#### Age and strategy influence

In PD patients, the receding late phase impairment on retest was paralleled by an improvement of strategy use. Specifically, patients' strategy scores, which were lower than controls' on test (19.7 ± 3.8 vs. 28.3 ± 4.7; *t*_34_ = 2.0, *p* = 0.05), became normal on retest (30.0 ± 3.8 vs. 25.6 ± 4.7; *t*_23_ = 0.6, p ns). The relationship between strategy scores and errors also evolved on the second session. Originally correlated with both search (*r* = −0.60, *p* = 0.01) and repetition errors (*r* = −0.55, *p* = 0.03), strategy scores became correlated solely with repetition errors (*r* = −0.57, *p* = 0.02, compared to *r* = −0.36, p ns, for search errors). Interestingly, comparison of controls' performance on test across age classes indicated that, like PD, normal aging preferentially affected the late learning phase (age < 60 years, *n* = 12 vs. age ≥ 60 years, *n* = 8: 2.5 ± 0.6 vs. 6.0 ± 1.4; *t*_18_ = 2.3, *p* = 0.04, for repetition errors, compared to 5.0 ± 0.5 vs. 7.5 ± 1.3; *t*_18_ = 1.7, *p* ns, for search errors).

#### Summary

PD disrupted both early and late learning of novel visuo-motor associations, but differentially so. The early deficit receded with additional training and associated improved strategy use, and was unrelated to disease stage. By contrast, the late deficit persisted despite additional training and better strategy use, and its magnitude was systematically correlated with disease stage as evaluated by Hoehn & Yahr on medication scores. In healthy subjects, late learning likewise seemed more vulnerable to aging than early learning.

## Discussion

The present study provides the first evidence that conditional learning of associations between visual stimuli and motor acts is impaired in PD patients. Early and late learning, respectively, preceding and following the first correct response, were both disrupted, but the late learning deficit was more closely linked to disease severity and more enduring across sessions than the early learning deficit. Given that caudal putamen is particularly affected in PD patients, this second finding provides some support to the idea that putamen-dependent habitual responses are more affected by PD than caudate-dependent goal-directed actions. These two points will be discussed in turn.

### Global conditional visuo-motor learning impairment in PD patients

Since Mishkin et al.'s (1984) proposal that habits depend on the striatum, the most convincing evidence supporting this claim came from studies using probabilistic tasks such as the weather prediction task (Knowlton et al., 1996; Frank et al., 2004; Shohamy et al., 2004a). By contrast, studies using conditional (motor or non-motor) learning tasks with error correction yielded inconsistencies (Gotham et al., 1988; Canavan et al., 1989; Vriezen and Moscovitch, 1990; Sprengelmeyer et al., 1995; Postle et al., 1997; Pillon et al., 1998; Marie et al., 1999). Here, by applying to PD patients the no-correction procedure used earlier in monkeys and healthy humans, we found a clear-cut learning impairment that endured over two testing sessions separated by a few hours. Two factors may have contributed to this positive finding in addition to the no-correction procedure.

The youth of a majority of our subjects is one of them: 12/20 controls and 9/16 patients indeed were under 60 years of age. Elderly subjects suffer from a decline in dopamine activity that impairs learning (Volkow et al., 1998; Floel et al., 2008) and habit tasks such as conditional learning are particularly sensitive to this normal aging effect (Levine et al., 1997; Schmitt-Eliassen et al., 2007). Had we included only our subjects over 60 years of age, we would have missed the disease effect as did Canavan et al. (1989) earlier, who compared young patients averaging 55 years of age to older controls averaging 60 years of age, that is, a pathological dopaminergic and striatal dysfunction to a physiological one. To what extent PD disrupts habit learning over and above normal aging is a question that remains to be addressed.

The second factor is that PD-induced brain damage is progressive, thereby leaving room for compensatory mechanisms. At the neural level, compensation varies with the task at hand. PD patients' normal performance in the weather prediction task is associated with an activation in temporal areas that is absent in controls (Moody et al., 2004), whereas normal performance in conditional visuo-motor learning is accompanied by greater activation in the prefrontal cortex relative to controls (Bedard and Sanes, 2009). At the behavioral level, within the present patients, those who resorted to the optimal learning strategy ended up learning normally, one patient even outperforming most controls. Thus, some early studies may have failed to detect conditional learning impairment in PD patients because of alternate strategies that went undetected.

The point that the present study does not help clarifying is the influence of PD patients' drug state on visuo-motor learning. Patients performed slightly but not significantly better after an acute challenge of levodopa than after an overnight withdrawal of medication. Several other recent studies (De Wit et al., 2011; Jocham et al., 2011; Shiner et al., 2012) also failed to find a direct effect of medication on PD patients' learning. These failures provide a reminder that above and beyond the well-known contribution of phasic dopamine to reward prediction errors, the neuromodulator role of dopamine in the brain is highly complex (see e.g., Wunderlich et al., 2012b for a recent discussion of this issue).

### Goal-directed vs. habit deficits in PD patients

In recent years, the learning theory literature has emphasized that what was classically viewed as a unique anatomo-functional system in the memory literature, habit learning, may in fact engage two distinct systems. In this theory, goal-directed actions and a “model-based” form of reinforcement learning are opposed to habitual responses and a “model-free” form of reinforcement learning (for review see Doll et al., 2012). One study tested patients with mild PD to distinguish the disease effect on the two processes embedded in habit learning (De Wit et al., 2011). In agreement with the present study, the authors found a global learning impairment that was not remediated by dopaminergic medication. Yet, in apparent contradiction with both the present study and the model, which predicted that patients should suffer from a disruption of habitual responses, patients displayed only a disease severity-dependent deficit in goal-directed actions.

In light of the present data, it is possible that a habit deficit did exist in the previous study but was masked due to normal aging decline in controls, compensatory strategy in patients, or both. All three groups in the previous study (on, off, and controls) were over 60 years of age. The deleterious effect of PD on habits might simply have been indistinguishable from that of normal aging in these elderly subjects. As regards strategy, patients in De Wit et al. (2011) did not use any explicit compensatory strategy. It nevertheless remains possible that some of them developed an implicit one. This appears even more likely given that disease duration and severity were mild (6–8 years of disease instead of 11 years in the present study, Hoehn & Yahr scores < rather than >2) and that we found that the lower the disease stage, the greater the tendency to use an implicit strategy.

This said, however, there is also an important methodological difference across the two studies that may explain the discrepancy between the results. Both studies aimed at evaluating goal-directed actions and habitual responses in PD patients, but they operationalized the concepts differently. The previous study was based on the theory that a habit is an instrumental response that persists even when its outcome is no longer valued. It measured conflict-induced habits, which surprisingly produce longer response times than goal-directed actions. This represents a major deviation from the training-induced habits measured by classical associative tasks, which result in quicker habitual responding. Thus, the diverging results of the two studies vis-à-vis habitual responses might simply due to the fact that they measure two different forms of habits.

Here, we studied classical training-induced habits as measured by conditional visuo-motor learning. We focused on two learning phases operationally defined as, respectively, encompassing trials and errors preceding vs. following the first correct response. We presumed that the early phase predominantly (though probably not exclusively) relies on goal-directed actions, while the late phase reflects the emergence of habitual responses, based on two lines of evidence. First, in monkeys, there exist two learning-related neural changes, one transient, frequent in the caudate nucleus, and occurring very early in learning; the other, durable, frequent in the putamen, and stabilizing late in learning (Hadj-Bouziane and Boussaoud, 2003; Hadj-Bouziane et al., 2006; Williams and Eskandar, 2006). Second, in healthy humans, the caudate nucleus and putamen subserve different computations during learning with, as in monkeys, the caudate nucleus more involved in early learning and the putamen more involved in late learning (Brovelli et al., 2011; Amiez et al., 2012). PD disrupted both the early and late learning of novel visuo-motor associations, in agreement with the fact that, at the advanced stages studied here, the disease interferes with both the associative and sensori-motor loops. However, late learning was more closely linked to the disease and more enduringly affected by it than early learning. First, only the magnitude of the late deficit was positively correlated with Hoehn & Yahr on medication scores. Second, only the late deficit persisted on the second testing session despite performance improvement and better strategy use.

The optimal learning strategy for the present task, repeating the same movement until it proves correct, is of more help during the search for the correct response than during its repetition and, hence, may more effectively reduce patients' goal-directed deficit than their habitual responses deficit. Strategy-related compensation, however, is probably not the sole factor explaining the differential effect of PD on the two learning phases. The sensori-motor loop remains more dysfunctional than its associative counterpart as the disease progresses (Redgrave et al., 2010). We studied earlier a group of PD patients similar to the present ones, that is, non-demented, levodopa-responsive subjects fulfilling criteria for surgical treatment and, accordingly found greater dopamine depletion in the putamen than in the caudate nucleus (Broussolle et al., 1999). The greater vulnerability of late learning to the disease effects observed here for visuo-motor associations could result from this more extensive putamen damage. This speculation is in agreement with the idea of a preferential involvement of the putamen in habitual responses.

Importantly, here, we could only assess the effect of PD on the emergence of habits. We did not test over-learned habits of the kind we all use in daily life when stopping at a red traffic light or speeding through a green one, and that require very long learning periods, incompatible with neuropsychological testing. Extensively trained automatic responses do engage the posterior putamen in healthy humans (Wunderlich et al., 2012a), but it remains to be proved that there are accordingly disrupted by the putamen progressive dysfunction produced by PD.

## Conclusion

The present sample of 16 early onset, non-demented, dopa-responsive patients suggests that when PD reaches moderate to severe stages it does interfere with habit learning as measured by conditional visuo-motor associative learning. This finding enriches the still meager body of data supporting the idea proposed by the memory literature that habits depend on the striatum like memories depend on the medial temporal lobe. The deficit following the first correct response seemed the most sensitive to the illness effects in the patient population tested in the present study. As Broussolle et al. (1999) found greater putamen than caudate dysfunction in similar patients, this finding is compatible with the recent view proposed by the learning theory literature that habits include two processes early, caudate-dependent, goal-directed actions and late, predominantly putamen-dependent, habitual responses. The patients' deficit was rarely dramatic, though. It was often compensated by optimization of the learning strategy, and was sometimes hardly distinguishable from normal aging decline. This showed that marked inter-individual variations occur even in a clinically quite homogeneous group.

### Conflict of interest statement

The authors declare that the research was conducted in the absence of any commercial or financial relationships that could be construed as a potential conflict of interest.
